# Global health educational trips: ethical, equitable, environmental?

**DOI:** 10.1136/bmjgh-2022-008497

**Published:** 2022-04-22

**Authors:** Lotta Velin, Kim Van Daalen, Renzo Guinto, Sibylle Herzig van Wees, Senjuti Saha

**Affiliations:** 1 Centre for Teaching and Research in Disaster Medicine and Traumatology, Department of Biomedical and Clinical Sciences, Linköping University, Linkoping, Sweden; 2 Cardiovascular Epidemiology Unit, Department of Public Health and Primary Care, University of Cambridge, Cambridge, Cambridgeshire, UK; 3 Planetary and Global Health Program, Saint Luke's College of Medicine William H Quasha Memorial, Quezon City, Philippines; 4 Sunway Centre for Planetary Health, Sunway University, Selangor, Malaysia; 5 Karolinska Institute, Stockholm, Sweden; 6 Child Health Research Foundation, Dhaka, Bangladesh

**Keywords:** Health education and promotion

Global health education in medical schools and at premedical undergraduate levels in high-income countries is often limited to short courses aimed at introducing students to the topic. These courses frequently include or focus on trips to low and middle-income countries, so the students can ‘experience global health*’*. There are several problems with this type of learning experience. To begin with, this form of ‘global health tourism*’* can be traced back to ‘tropical medicine*’*—a field plagued by paternalism and asymmetric power dynamics, where the so-called ‘Global North’' dominated the discourse at the expense of the ‘Global South’. Such ‘global health tourism*’* supports the frequent misinterpretation of global health as ‘*health abroad”* 1. More recent conceptualisations of global health also include local health, although shaped by global forces, with emphasis on marginalised communities and achieving health equity and justice for all^1 2^. In the wake of recent calls to decolonise global health^2–6^ and critical reflections of the current field of practice^7–9^, and the lifting of COVID-19 travel restrictions within many academic institutions, there is an imperative to reassess global health educational trips organised or initiated by high-income country (HIC) institutions from an ethical perspective. Meanwhile, due to the worsening climate crisis and the emergence of the concept of planetary health—a solution-orientated transdisciplinary field that envisions a healthy future for both humanity and the earth’s natural systems on which it depends^10^. Likewise, there has been a growing discourse around limiting work-related travel in global health education and academia^11^. However, while global health education and academia have been critically discussed in recent years, ecological perspectives, which innately are intertwined with ethics and equity, have not been integrated in the conversations. Therefore, we propose a checklist with six guiding questions to support the assessment of whether a global health educational trip initiated or promoted by a higher education institution in a HIC is appropriate from an ethical, equitable and environmental perspective (figure 1).

**Figure 1:**
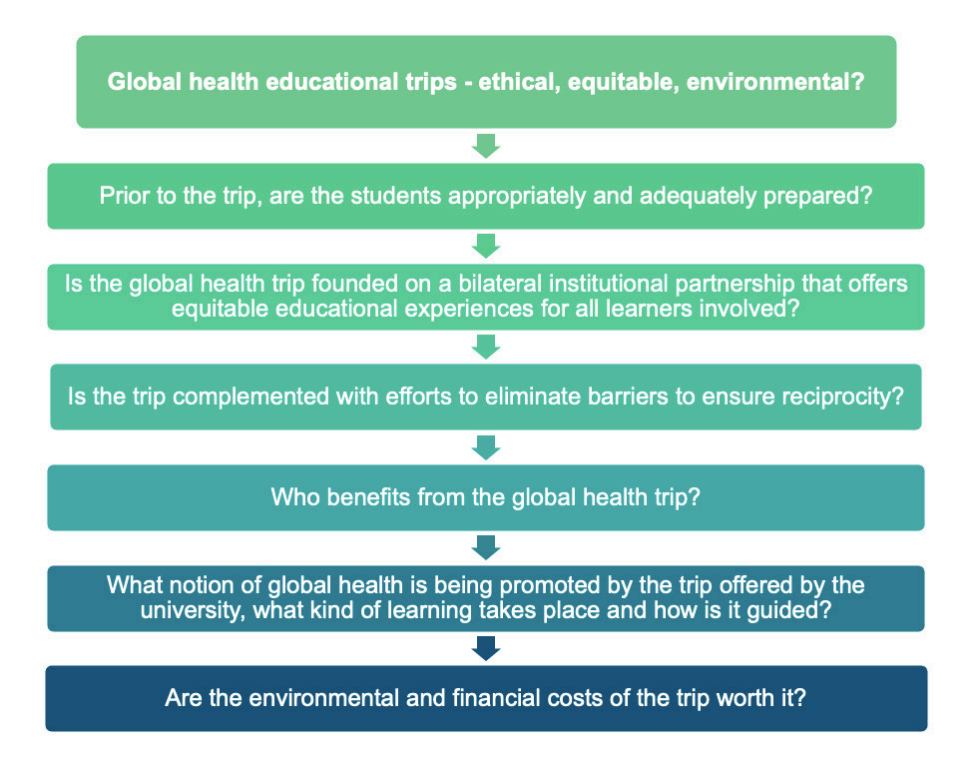
The proposed checklist with six guiding questions to help assess whether a planned global health educational trip should be conducted, considering ethical, equitable and environmental perspectives.

This checklist is not intended as a comprehensive resource but rather provides a set of critical guiding questions for educators and faculty involved in global health curriculum design to support the reflective process of designing their global health programmes. Each question is subsequently followed by an explanation of why this question is relevant, building on the published work in ethical global health of many global health educators and experts. 6 12 13

First, prior to the trip, are the students appropriately and adequately prepared? This question is important because predeparture training allows for engagement with the necessary theoretical and practical knowledge required for ethical trips. The syllabi for the first segments of global health courses should include critical analysis and reflection of ideas like ‘decolonialism’ and ‘true bidirectionality’^13^. Decolonialism is defined as a movement for critical, anticolonial reflection to ‘repoliticise’ and ‘dehistoricise’ global health^2^. Bidirectionality can be defined as equitable educational experiences for learners from both the low and middle-income countries (LMIC) and HIC institutions. It may be argued that such terms have become buzzwords, but the original definitions are grounded in principles of justice and reject the inherited postcolonial power imbalances portrayed in global health today.

Second, is the global health trip founded on a bilateral institutional partnership that offers equitable educational experiences for all learners involved? Bilateral exchanges based on shared models of learning empower students to become agents of change in their local contexts by broadening their perspectives on justice and equity, increasing cultural and linguistic competence and stimulating motivation to work with underserved marginalised populations^14 15^. Students who have participated in these experiences may further strengthen and sustain the partnership and enhance mutual understanding of health systems and cultures between institutions^14^. While educational trips are often a collaboration between HIC and LMIC institutions, there is rarely parity in the partnership. Illustratively, few LMIC students participate in educational trips to HIC institutions or other LMICs. Equity at the most basic level may mean equal numbers of students from each institution benefiting from the exchange, but other mechanisms should be considered to ensure that the total benefits, as perceived by both parties, are of equitable worth^16 17^. This could mean that a HIC institution has the opportunity to send students to an LMIC, and that the LMIC institution receives other benefits in return, for example, educational and academic opportunities, collaborations or resources. These other forms of benefits should be agreed on based on the LMIC institution’s indication of what is considered meaningful. Similarly, potentially negative consequences of educational trips must be prevented and mitigated to ensure that the partnership is bilaterally beneficial. This may include efforts to alleviate the disproportionate time investment host institutions bear due to logistic support and interruptions to clinical care when LMIC students/teachers host visiting HIC students or go on corresponding trips to HICs.

Third, is the trip complemented with efforts to eliminate barriers to ensure reciprocity? This question is important as multiple barriers often hinder LMIC stakeholders from accessing the intended bidirectional benefits of HIC partnerships. The partnership should ensure to address such barriers to reciprocity, including (1) a lack of financial resources that may limit LMIC students/faculty from going to HIC institutions, (2) potential income loss that may be experienced during the time spent away from day-to-day occupations and (3) restrictive, complex and costly visa policies that disproportionally limit LMIC affiliates from travelling abroad for educational trips, courses, research fellowships or scientific conferences.^9^ Financial, logistical and, if needed, legal support should be made available and integrated into the planning of educational trips to ensure that all students/faculty can equitably participate in the partnership.

Fourth, who benefits from the global health trip? A student exchange should not only benefit the students who travel and their personal and professional development. When considering this question, the impact of the exchange should also involve possible benefits to their home institution with the novel clinical and educational approaches learnt and advantages through further collaborations developed during the immersion (eg, the establishment of research projects). Opportunities to ensure benefits also for the broader field of global health and their local communities, respectively, should also be considered, for example, by knowledge cocreation through research or implementation of interventions, resulting in improvement in health outcomes and determinants.

Fifth, what notion of global health is being promoted by the trip offered by the university, what kind of learning takes place and how is it guided? Educational trips to LMICs have traditionally promoted the notion of global health as ‘foreign health*’*, or more precisely, ‘poor countries’ health*’*. Instead, we recommend that global health teaching and associated trips should focus on holistic perspectives beyond geographical boundaries^1^. This includes covering content on colonialism, power structures, privilege, racism, health equity and justice. This can be achieved through prioritising and respecting voices, knowledge and experiences from people living within affected communities, LMICs and indigenous academics 13 and actively acknowledging that HICs have much to learn from the health expertise of LMICs.

Finally, are the environmental and financial costs of the trip worth it? Educational trips are not free—neither financially nor environmentally. Considering the brevity of such trips, institutions must appraise if the benefits are worth the cost and whether the available resources could be spent differently to develop sustainable partnerships. In addition to the financial costs, the environmental toll of international travel is well recognised. Contributions to further green house gas emissions during the ongoing climate crisis that are impacting human health globally^18^ and exacerbating multidimensional health inequities within and between countries should be considered. For instance, a return trip for one person to travel by plane between London-Delhi and Boston-Kigali can be estimated to ‘cost’ 1.98 versus 3.11 tonnes of carbon dioxide equivalents^19^. These are numbers far exceeding the annual carbon footprint per capita in some of the poorest sub-Saharan African countries such as Chad and Niger, which is around 0.1 tonnes^20^. Climate change and its causes reveal deeper notions of injustice and ‘environmental racism’^21^. The costs of climate change are disproportionately affecting LMICs who bear the brunt of its consequences, including the health-related impacts—while having lesser means to adapt to and mitigate the consequences of the changing realities^22^. This despite most LMICs having very limited historical contribution and responsibility for these emissions—in contrast with HICs such as the USA and the European Union, which account for 25% and 22% of emissions since 1751, respectively^23^. Those that are contributing the least are being impacted the most. Such environmental power imbalances should be considered when designing global health educational trips to ensure that the ‘do no harm’principle is also embraced from an ecological perspective.

These guiding questions do not undermine the benefit of some international travel in global health education. If a trip is considered ‘essential’, then air travel and other environmental costs for student exchanges should be offsetted through accredited schemes. Such schemes often include investments in environmental projects in LMICs, however, caution must be taken to ensure that such offsettings entail benefits that would not have been acquired otherwise (eg, an already planned clean-energy project cannot be counted as offsetting)^24^. Such investments should ideally be planned together with the LMIC institutions. Moreover, the hiatus in international travel during the COVID-19 pandemic opened new ways of global health teaching^13^. Online interactions have grown immensely, offering new opportunities for collaborative teaching such as inviting LMIC guest lecturers, holding joint sessions between LMIC and HIC institutions and organising ‘virtual’ field visits. Once global health trips resume—these different teaching innovations must remain as more ethical, equitable and environmental alternatives.

We recognise that we are writing from a position of privilege, as we are cognisant that travelling abroad has been a key element in our individual journeys into global health. Yet, it is time for our collective global health community to become more mindful and intentional in our actions, including our travels, in the spirit of equity, decolonialism and planetary health. Furthermore, while this checklist is a tool for critical self-examination targeting primarily HIC-organised trips, we envision that global health educational trips become available equitably to all kinds of students and early career professionals, whether from HICs or LMICs. Power should not remain solely in the hands of educators at HIC institutions; educators and faculty from LMICs institutions should be meaningfully and equitably involved, and HIC actors should take their active responsibility to dismantle historical power dynamics. Ultimately, how we teach global health frames the way we see (and later, practice) global health. Introductory global health education must not only teach but be founded on and demonstrate equity and justice; the core concepts of our field. This requires not just changing the way we travel, but a true integration of local and indigenous perspectives rather than only looking at global health as ‘health somewhere else’ 1.

## Data Availability

There are no data in this work.
